# Progression-free survival as a surrogate for overall survival in oncology trials: a methodological systematic review

**DOI:** 10.1038/s41416-020-0805-y

**Published:** 2020-03-26

**Authors:** Lisa Belin, Aidan Tan, Yann De Rycke, Agnès Dechartres

**Affiliations:** 1 Sorbonne Université, INSERM, Institut Pierre Louis d’Epidémiologie et de Santé Publique, AP-HP, Hôpitaux Universitaires Pitié Salpêtrière - Charles Foix, Département Biostatistique Santé Publique et Information Médicale, F75013 Paris, France; 2 Preventive Medicine, National University Hospital, Singapore, Singapore; 3 INSERM, Institut Pierre Louis d’Epidémiologie et de Santé Publique, AP-HP, Hôpital Pitié-Salpêtrière, Hôpitaux Universitaires Pitié Salpêtrière - Charles Foix, Département Biostatistique Santé Publique et Information Médicale, Centre de Pharmacoépidémiologie (Cephepi), F75013 Paris, France

**Keywords:** Cancer, Outcomes research

## Abstract

**Background:**

Progression-free survival (PFS) is a surrogate endpoint widely used for overall survival (OS) in oncology. Validation of PFS as a surrogate must be done for each indication and each intervention. We aimed to identify all studies evaluating the validity of PFS as a surrogate for OS in oncology, and to describe their methodological characteristics.

**Methods:**

We conducted a systematic review by searching MEDLINE via PubMed and the Cochrane Library with no limitation on time, selected relevant studies and extracted data in duplicate on how surrogacy was evaluated (meta-analytic approach, assessment of correlation and level of evaluation).

**Results:**

We identified 91 studies evaluating the validity of PFS as a surrogate for OS in 24 cancer localisations. Although a meta-analytic approach was used in 83 (91%) studies, the methods used to validate PFS as a surrogate of OS were heterogeneous across studies. Of the 47 studies concluding that PFS is a good surrogate for OS, for 15 (32%), there was no quantitative argument for surrogacy.

**Conclusions:**

Although most studies used a meta-analytic approach as recommended, our methodological review highlights heterogeneity in methods and reporting, which stresses the importance of developing and applying clear recommendations in this area.

## Background

According to the Biomarkers Definition Working Group, a surrogate endpoint is a biomarker intended for substituting a clinical endpoint and expected to predict clinical benefit, harm or lack of these.^1^ Surrogate endpoints are used for measuring treatment benefits earlier, and allowing for a lower sample size. They should have a scientifically plausible link to the true endpoint, and ideally, the surrogate should be embedded within the causal pathway of the disease progression to the true endpoint.^2^

In oncology, surrogate endpoints, especially progression-free survival (PFS), are frequently used as primary endpoints in place of overall survival (OS), the most clinically meaningful endpoint. More than one-third of trials that received US Food and Drug Administration (FDA) marketing approval between 2009 and 2013 used PFS as a primary endpoint,^3^ which is possible in theory only if PFS has been validated as a surrogate endpoint for OS. Otherwise, approvals may be misleading^4^ as underlined by the example of bevacizumab that obtained FDA-accelerated approval in 2008 for metastatic breast cancer based on PFS improvement.^5^ Approval was withdrawn in 2011 after several trials showed that the PFS improvement was lower than expected, with no OS improvement.^6^

Surrogate validation is a demanding procedure that must be performed for each clinical context (localisation, indication and treatment). For years, surrogacy and correlation have been confused.^7^ Methods for surrogacy validation have evolved over recent decades. Prentice, in 1989, was the first author to introduce four criteria to be met to support surrogacy: (1) treatment has a significant impact on the surrogate endpoint; (2) treatment has a significant impact on the true endpoint; (3) the surrogate and true endpoints are correlated; (4) the full effect of treatment on the final endpoint is captured by the surrogate. Four major frameworks have been developed for evaluating surrogate markers in randomised trials.^8^ In this study, we focused on the “causal-association” paradigm in which the effect of treatment on the surrogate is associated, across studies or population subgroups, with its effect on the true endpoint. This paradigm includes the meta-analytic method, which evaluates the association between the surrogate and the true endpoint across individuals, as well as a correlation of the treatment effect between both endpoints across studies. This meta-analysis method has become the recommended approach.^9^ Both questions are of interest: the first for patient management (the surrogate being a marker of prognosis), and the second for drug development (use of the surrogate potentially gaining months or years of development time). Other criteria proposed to assess the validity of surrogate endpoints include IQWIG^10^ or the biomarker surrogate evaluation schema (BSES).^11^

In this study, we aimed to systematically review all studies evaluating the validity of PFS as a surrogate for OS in cancer clinical trials, with a particular focus on the methods used and whether they were appropriate.

## Methods

We performed a methodological review of studies evaluating the validity of PFS as a surrogate for OS in cancer clinical trials.

### Search strategy

We searched for all studies evaluating the validity of PFS as a surrogate for OS in cancer clinical trials published as abstracts or in full in MEDLINE via PubMed, in the Cochrane Central Register of Controlled Trials and in the Cochrane Methodology Register with no restriction on time. A search algorithm was developed, including specific free-text words and relevant keywords related to cancer and to PFS and surrogate endpoints (Supplementary Information 1). We also searched MEDLINE via PubMed for authors frequently involved in surrogacy studies. Finally, we screened the reference lists of identified citations and relevant systematic reviews in the field. The search was first conducted on November 30, 2017 and updated on April 1, 2019.

### Eligibility criteria

We included all reports of studies evaluating the validity of PFS as a surrogate for OS in cancer clinical trials, whatever the evaluated treatment, tumour location, type of study and statistical methods used. Studies documenting PFS and OS as two independent endpoints without evaluating the association between these two endpoints were not eligible. We excluded studies not in the field of oncology and not in therapeutic evaluation, and those evaluating the extrapolation of pharmacodynamics properties to clinical endpoints. We also excluded studies evaluating the validity of surrogate endpoints other than PFS, such as biomarkers, tumour growth, disease-free survival or time to progression. Letters were excluded because quantitative results could not be extracted. Abstracts of studies subsequently reported in full were excluded. When the selected study was a collection of meta-analyses, we included each meta-analysis individually. When the same data were analysed several times with different methods, we selected the first publication.

### Selection process

Two reviewers (L.B. and A.T.) independently selected potentially relevant references according to the prespecified eligibility criteria by first assessing the title, abstract and full text whenever necessary. All disagreements were resolved by discussion with a third reviewer (A.D.) to reach consensus.

### Data extraction

A standardised form was developed and tested on a sample of five studies. The following data were collected independently by two reviewers (L.B. and A.T.) from the reports of studies, including appendix, online supplements and errata:

- Publication characteristics: year of publication and journal name. We classified journals as general medical, oncology or epidemiology/biostatistics. We stated whether a funding source was reported and the nature of this source. We noted whether epidemiologists or statisticians were involved as authors according to the definition reported by Delgado-Rodriguez et al.^12^
- Clinical characteristics: population (adults or children, localisation of cancer and advanced or localised cancer), and interventions evaluated (immunotherapy, targeted therapy, conventional chemotherapy, radiotherapy and surgery). When several interventions were investigated, we extracted the interventions evaluated for the primary analysis of surrogacy.
- Definition of PFS: definition of tumour progression (according to Response Evaluation Criteria in Solid Tumors [RECIST] criteria,^13^ World Health Organization criteria and Response Assessment in Neuro-Oncology (RANO) criteria) and timeframe. When both PFS and time-to-progression (TTP) data were available, we extracted PFS data. When authors used PFS or TTP data indiscriminately, we used PFS/TTP data as PFS data.
- Methodological characteristics: we stated whether the study was based on a collection of meta-analyses, a single meta-analysis, a collection of trials or a single-trial analysis. We noted whether meta-analyses were based on aggregated or individual patient data. We gathered information on which methods were used to evaluate surrogacy: correlation coefficient (r), determination coefficient (R²), surrogate threshold effect (STE)^14^ and Kendall’s τ. The explanation of these parameters is detailed in the paragraph below.
- Results: we collected all results related to surrogacy evaluation (r, R² and STE). For each result, we stated that it was estimated at the individual or the trial level. In the latter case, we extracted whether the result was the correlation of aggregated measures (e.g., correlation between the median value of PFS and OS in both groups) or treatment effects (e.g., correlation between the hazard ratio of PFS and OS).
- Conclusions of the authors. We extracted the conclusions of the authors, which we classified into three categories:
  - Validation: “PFS is a valid surrogate endpoint for OS”,^15^ “our analyses also show that PFS is a good surrogate for OS”.^16^
  - Partial validation: “PFS was moderately associated with OS”,^17^ “modestly correlated with improvement in OS”.^18^
  - No validation of surrogacy: “did not correlate sufficiently with OS to be used as a surrogate endpoint”,^19^ “results suggest that the surrogacy could not be confirmed”.^20^

All disagreements between the two reviewers (L.B. and A.T.) were discussed to reach consensus.

### Statistical tools and methodology of surrogacy validation

Many statistical methods have been developed.^2^ A meta-analytic approach has been recently strongly recommended,^21^ and individual patient data (IPD) meta-analysis is the only design allowing an evaluation at both the patient and trial levels. The statistical methods that can be used are

- Patient-level evaluation: assessed using patient-level data (from an IPD meta-analysis or a single-trial analysis).
  - Assessing the correlation between the potential surrogate endpoint (PFS) and the final endpoint (OS) in the whole trial using a Pearson’s correlation coefficient (r). This is a simple and reductive approach, because it provides a surrogacy evaluation at the patient level without taking into account the treatment arm. Several studies warned against this simplistic approach.^2,21^
  - In the context of time-to-event endpoints, the two endpoints (PFS and OS) can be jointly modelled to estimate a correlation parameter, reflecting the association between the candidate surrogate and the final endpoint (OS), regardless of the treatment arm.

- Trial-level evaluation: It could be assessed based on aggregated data or IPD meta-analyses with two main approaches:
  - In the first approach, the treatment effects on PFS and OS are estimated separately using hazard ratios (HR). The association between both treatment effects is then assessed by the coefficient of determination R² of a linear regression model, usually weighted by trial size. This approach fails taking into account estimation error.
  - The second approach follows the two-stage model introduced by Burzykowski and Buyse.^9,22^ In the first stage, the treatment effects are estimated simultaneously using a bivariate model. The association between both treatment effects is then estimated using an error-in-variable model, to adjust for estimation errors, and the coefficient determination R² is estimated.

With both approaches, it is possible to estimate the surrogate threshold effect (STE) defined as the minimum treatment effect on the surrogate necessary to predict a non-zero effect on the true endpoint.^13^

### Evaluation of surrogacy validation

As previously stated, several criteria have been proposed to assess the validity of surrogate endpoints (IQWIG,^10^ biomarker surrogate evaluation schema (BSES)).^11^ Although these approaches present differences, they all require a trial-level correlation ≥ 0.6 to definitely validate a surrogate endpoint. BSES focused on the coefficient of determination R² to qualify surrogacy as “poor”, “fair”, “good” or “excellent”, whereas IQWIG used correlation measures without detailing the statistical tools used (coefficient of correlation and coefficient of determination).

**Fig. 1:**
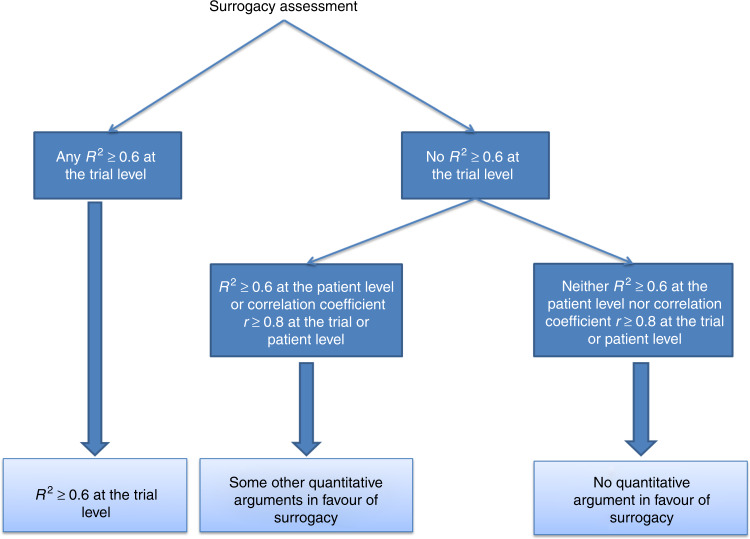
Definition of surrogacy assessment evaluation.

For each study, we evaluated the surrogacy based on extracted quantitative results, and by using a conservative approach, we considered that PFS was a valid surrogate when the authors reported *R*² ≥ 0.6 estimated at the trial level. We considered that PFS could be a surrogate with *R*² < 0.6 at the trial level if other correlation parameters were ≥0.8 whatever the level considered (trial or patient). We considered that PFS was not a surrogate when neither the determination coefficient *R*^2^ was ≥0.6, nor any correlation parameters were ≥0.8 at trial or patient level (see Fig. 1 for detailed definition). We compared this evaluation with the conclusions on surrogacy provided by the authors.

### Statistical analyses

The analysis was mainly descriptive. Qualitative variables are described with frequencies, and quantitative variables with median (Q1–Q3). Analyses were performed with R v3.3.2.

## Results

### Selection

**Fig. 2:**
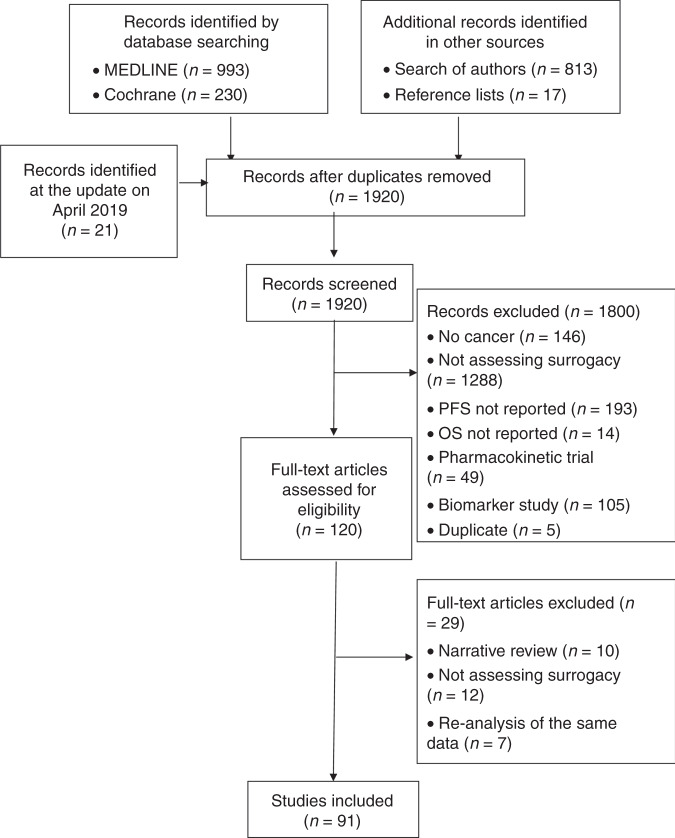
Flow of the selection of articles.

After screening 1920 references retrieved by the search, we included 91 studies (Fig. 2; Supplementary Information 2 for the list of included studies).

### General and clinical characteristics

Study characteristics are presented in Table 1. Half of the selected studies were published after 2015. The most frequent cancer localisations investigated were lung cancer in 20 (22%) studies, colorectal cancer in 15 (17%) and breast cancer in 13 (15%). Six (7%) studies pooled many cancer localisations in the same surrogacy study. In 90 (99%) studies, PFS surrogacy was evaluated in advanced cancer. The only study that did not concern advanced cancer included resectable oesophageal cancers. In the primary analysis of the selected studies, the investigated treatment was chemotherapy in 81 (89%) studies, targeted therapies in 50 (55%) and immunotherapy in 19 (21%). Immunotherapy was more frequent in studies published after 2017 (35% after 2017 vs. 15% before 2017). As described in Supplementary Information 2, combinations of treatment were investigated in most studies.

**Table 1:** Characteristics of included studies (*N* = 91).

*Source of study*	
Journal article	87 (96%)
Conference abstract	4 (4%)
*Type of journal*	
Oncology journal	75 (86%)
General journal	11 (13%)
Epidemiology or biostatistics journal	1 (1%)
*Biostatistician involved*	41 (45%)
*Funding*	
No specific funding	53 (58%)
Public funding	20 (22%)
Private funding	15 (17%)
Public and private funding	3 (3%)
Date of publication, median (Q1–Q3)	2015 (2013–2017)
*Type of study*	
Meta-analysis on aggregated data	65 (71%)
Meta-analysis on individual patient data	18 (20%)
Trial	8 (9%)
*Population*	
Adults	91 (100%)
*Localisation*	
Lung cancer	20 (22%)
Colorectal cancer	15 (17%)
Breast cancer	13 (15%)
Haematological cancer	6 (7%)
Several localisations	6 (7%)
Gastric cancer	5 (5%)
Pancreas cancer	5 (5%)
Head and neck cancer	4 (4%)
Renal cancer	4 (4%)
Other cancer	13 (14%)
*Stage of cancer*	
Advanced	77 (85%)
Localised	1 (1%)
Both	13 (14%)
*First-line treatment*	
Yes	32 (35%)
No	13 (14%)
Both	44 (49%)
Not reported	2 (2%)
*Type of treatment*	
Chemotherapy	81 (89%)
Targeted therapy	50 (55%)
Immunotherapy	19 (21%)

PFS was defined as the time elapsed between a time origin and the occurrence of progression or death in 89 (98%) studies. The origin timepoint was reported for 43 (47%) studies. The definition of progression was reported for 31 (34%) studies. In solid tumours, when the definition of progression was reported, RECIST criteria were used in 28/29 (97%) studies.

### Methodological characteristics

Most studies were meta-analyses (*n* = 83), based on aggregated data in 65 (78%) and individual patient data in 18 (22%). The methods used to assess the surrogacy were heterogeneous among studies (Table 2).

**Table 2:** Study design and methods used to evaluate the surrogacy of progression-free survival for overall survival.

	Meta-analysis on aggregated data (*N* = 65)	Meta-analysis on individual patient data (*N* = 18)	Trial (*N* = 8)	**All (*N*** = **91)**
*Evaluation of correlation at patient level*	NA	14 (78%)	8 (100%)	22 (24%)
Coefficient of correlation	NA	11 (61%)	7 (88%)	18 (20%)
Coefficient of determination R²	NA	3 (17%)	5 (63%)	8 (9%)
*Evaluation of correlation at trial level*	64 (98%)	15 (83%)	NA	79 (87%)
Evaluation on aggregated measures^a^	37 (57%)	2 (11%)	NA	39 (43%)
Coefficient of correlation	31 (48%)	0 (0%)	NA	31 (34%)
Coefficient of determination R²	14 (22%)	1 (6%)	NA	15 (16%)
Tau de Kendall	0 (0%)	1 (6%)	NA	1 (1%)
Evaluation on treatment effect	50 (77%)	14 (78%)	NA	64 (70%)
∙ Hazard ratio	36 (55%)	14 (78%)	NA	50 (55%)
∙ Difference of median	13 (20%)	0 (0%)	NA	13 (14%)
∙ Ratio of median	1 (2%)	0 (0%)	NA	1 (1%)
Coefficient of correlation	29 (45%)	1 (6%)	NA	30 (33%)
Coefficient of determination R²	36 (55%)	13 (72%)	NA	49 (54%)
Surrogate threshold effect	11 (17%)	8 (44%)	NA	19 (21%)

*NA* not applicable.

^a^Median progression-free survival, the progression-free survival rate at a clinically meaningful timepoint.

#### Evaluation at the patient level

Patient-level correlation was evaluated in 22 (24%) studies, with a correlation coefficient in 18 and a determination coefficient in 8. Patient-level correlations were evaluated on 14 IPD meta-analyses and 8 single-trial analyses.

#### Evaluation at the trial level

Trial-level correlation was assessed in 79 (87%) studies, between aggregated measures of PFS and OS in 39, and between treatment effects on PFS and OS in 64. Of the 39 studies evaluating aggregated measures, correlation coefficients were used in 31, and coefficient of determination was used in 15.

Of the 64 studies evaluating treatment effect correlation, authors used hazard ratio to quantify the treatment effects in 50 studies. Correlation coefficients were used in 30 studies, whereas determination coefficients were used in 49 studies. Determination coefficients were obtained using a weighted linear regression model in 44 studies. Surrogacy threshold effect (STE) was used in 19 studies (21%).

Correlation was investigated at both the patient and trial levels in 11 (12%) studies, and all of them were IPD meta-analyses. Surprisingly, three IPD meta-analyses do not evaluate the surrogacy at trial level.

### Evaluation of surrogacy validation

Overall, 47 (52%) studies concluded on the validation of PFS as a surrogate for OS; for 24 (51%), a trial-level *R*² ≥ 0.6 was reported; for 8 (17%), other quantitative arguments favouring surrogacy without reaching *R*² ≥ 0.6 at the trial level were reported; for 15 (32%), no quantitative argument to conclude on surrogacy was reported (Table 3).

**Table 3:** Consistency between authors’ conclusions on progression-free survival surrogacy and quantitative arguments in favour of surrogacy according to the type of study.

Authors’ conclusions on surrogacy	*R*² ≥ 0.6 at trial level	Other quantitative arguments in favour of surrogacy	No quantitative arguments
*Individual patient data meta-analysis*			
Validation of surrogacy	8 (73%)	1 (9%)	2 (18%)
Partial validation of surrogacy	0 (0%)	0 (0%)	0 (0%)
No validation of surrogacy	1 (14%)	1 (14%)	5 (71%)
*Aggregated data meta-analysis*			
Validation of surrogacy	16 (48%)	7 (21%)	10 (30%)
Partial validation of surrogacy	3 (25%)	0 (0%)	9 (75%)
No validation of surrogacy	2 (10%)	0 (0%)	18 (90%)
*Single-trial analysis*			
Validation of surrogacy	NA	0 (0%)	3 (100%)
Partial validation of surrogacy	NA	0 (0%)	2 (100%)
No validation of surrogacy	NA	1 (33%)	2 (66%)

Discrepancy rates between authors’ conclusion and our evaluation of surrogacy validation based on reported quantitative arguments (see Fig. 2) are 27% for IPD meta-analyses, 49% for aggregated meta-analyses and 75% for single-trial analyses (Table 3). The agreement between quantitative arguments and the authors’ conclusions seemed better for IPD meta-analyses, followed by aggregated data meta-analyses and finally by single-trial analyses.

## Discussion

This systematic review identified 91 studies evaluating the validity of PFS as a surrogate for OS in 24 cancer localisations. Validation of the surrogacy of PFS was achieved by using heterogeneous methods. Only half of studies concluded on the validity of PFS as a surrogate for OS. However, among these, for 15, this conclusion was not supported by the results, with no quantitative argument in favour of surrogacy given.

Our study provides a complete overview of studies evaluating surrogacy validity for PFS, the most commonly used surrogate for OS in oncology, whatever the study design used. Consistent with previous studies,^22–25^ some studies used sophisticated methods specifically developed for surrogacy validation,^9,14^ whereas others had poor methodology (e.g., showing that PFS is significantly associated with OS, assessing a simple correlation between PFS and OS duration in a single-trial analysis without accounting for censoring, pooling studies with different control groups…).

Our work goes beyond the previous literature on the topic by providing the most up-to-date evidence on studies evaluating the surrogacy of PFS. A previous review^23^ on the topic was conducted before July 2016, before the development of such studies in the field of immunotherapies, which represent a major shift in cancer treatments. Indeed, the mechanisms of action of immunotherapeutic agents markedly differ from those of chemotherapy,^26^ which reinforces the need to evaluate PFS as a surrogate endpoint for OS in this therapeutic area. Most immunotherapy trials used PFS defined as the time from randomisation to progression according to the RECIST criteria or to death. The RECIST criteria were originally based on experience with cytotoxic agents. Therefore, uncertainty exists as to whether progression evaluated with RECIST criteria can sufficiently reflect the antitumour effect of these drugs. Two recent reviews^27,28^ focusing on immune checkpoint inhibitors concluded insufficient data to support PFS as a surrogate endpoint for OS. In our review, 19 studies evaluated immunotherapy: 8 concluded that PFS was a good surrogate (2 concerned haematological cancers, 2 renal cancers and 1 each soft-tissue sarcoma, colorectal cancer, gastric cancer and pancreatic cancer). Three of these eight studies reported R² ≥ 0.6, and three had other quantitative arguments to conclude on surrogacy. Recent approvals of pembrolizumab in lung cancer, nivolumab combined with ipilimumab in colorectal cancer or atezolizumab in triple-negative breast cancer will provide more data to evaluate the surrogacy of PFS in this innovative clinical context.

A mapping of the localisations and treatments for which PFS has been validated as a surrogate for OS would help in planning future trials. Unfortunately, this mapping is currently not possible because of the heterogeneity of methods used. For example, for non-small-cell lung cancer, of the four studies investigating the surrogacy of PFS for chemotherapy, three concluded that PFS was not a relevant surrogate, but the only study that concluded on PFS as a valid surrogate was a meta-analysis of individual patient data (the most adequate method to validate a surrogate).

Despite the recommendations by Buyse et al.,^2^ we found no evidence of improvement in surrogacy evaluation and reporting. This result could be explained by difficulties in applying recommendations such as IQWIG^10^ or BSES.^11^ These recommendations require confidence intervals and STE to validate the surrogacy. Consistent with previous studies,^23,29^ we found these items poorly reported. Because of the different recommendations, authors have difficulty choosing the right method to assess surrogacy. Our study also identified 15 (32%) studies for which the conclusion of surrogacy was not supported by the results, with no quantitative argument given for surrogacy (defined as no R² ≥ 0.6 or no correlation ≥ 0.8, whatever the level considered), and the rate of discrepancies differs according to the type of study. When authors used IPD meta-analysis, the results seem to be more consistent, whereas when surrogacy is established from a single-trial analysis, the results must be taken with caution. We also found eight studies that validated PFS as a surrogate with arguments other than the criteria we chose; seven studies reported a correlation coefficient ≥ 0.8 at the trial level, and one study reported a correlation coefficient ≥ 0.8 at the patient level. Our criteria focusing on whether or not the coefficient determination was ≥0.6 at the trial level may be too stringent, but at the same time, our criteria could be considered relaxed surrogacy criteria because three studies concluded no validation of PFS as a surrogate endpoint, but met our criteria. As well, we considered that the coefficient of determination calculated on aggregated measures at the trial level or between treatment effects was interchangeable. To our knowledge, there is no proof that one is better than the other for evaluating surrogacy.

Thus, simple recommendations on both methodology and reporting in this area are urgently needed, as previously claimed.^30^ A task force involving methodologists and oncologists would be necessary to propose recommendations to standardise criteria for surrogacy evaluation. To improve the reporting of the literature around surrogacy studies, and allow for a mapping of PFS surrogacy, a reporting guideline must also be proposed. The RESEEM statement has been recently published,^31^ and could be an answer to this issue. Authors present recommendations on how to report a surrogacy validation study, and help the investigators choosing the right method advocating for IPD meta-analysis.

Our study has several limitations. Because of the heterogeneity in recommendations (IQWIG and BSES), we chose a simple consensual criteria for surrogacy evaluation, so the frequency of inappropriate conclusions on PFS surrogacy (32%) could have been underestimated. However, it is important to note that surrogacy validation is not as simple as a binary classification. Statistical tools are a way to quantify the surrogacy between PFS and OS, but other arguments must be considered. As pointed out by Prentice et al.,^32,33^ causal context must also be considered, including biological or physiopathological rationale, mode of action of the therapy or whether there is an established surrogacy in closely related pathologies.

Because our study focused on published studies, we cannot exclude that our results may be affected by publication bias: trials with negative results being more likely to remain unpublished and thus excluded from surrogacy measures.^34,35^ Inferring the impact of taking into account unpublished trials on the estimated surrogacy measures is difficult. Although we only selected studies evaluating PFS surrogacy, some of them were meta-analyses, including some trials evaluating TTP or PFS indistinctively. As the aim of the meta-analysis was to evaluate PFS surrogacy, we have kept it in our sample that could introduce some heterogeneity. Finally, we did not evaluate the length of post-progression survival or the possibility of crossover. However, these elements have been found to influence the level of surrogacy.^22^

Our work provides a complete overview of studies evaluating the surrogacy of PFS on OS, and emphasises the heterogeneity in methods used to assess surrogacy. We also identified some studies for which the conclusion on validity of PFS was not supported by the results, suggesting that interpretation of the results of these studies should be careful. All these elements reveal the importance of developing clear recommendations about methods and reporting for surrogacy validation.

## Supplementary information

Supplementary Material

## Data Availability

Data will be available by request to the authors.
