# Supplementary material for: Progression-free survival as a surrogate for overall survival in oncology trials: a methodological systematic review
Source: Br J Cancer. 2020 Mar 26;122(11):1707–14. doi: 10.1038/s41416-020-0805-y (PMC7250908; doi:10.1038/s41416-020-0805-y)
Supplement: Supplementary file 1 — Supplementary Material [file 41416_2020_805_MOESM1_ESM.docx]

**Supplementary Information**

Supplementary Information 1: Search algorithm

1 “overall survival” [tiab]

2 “progression free survival “ [tiab]

3 “progression-free survival” [tiab]

4 disease free survival [mh]

5 disease progression [mh]

6 surrogate [tiab]

7 surrogacy [tiab]

8 1 OR 2 OR 3 OR 4 OR 5

9 6 OR 7

10 1 AND 8 AND 9

Supplementary Information 2: Selected studies

| **First author (year)** | **Localization of cancer** | **Stage of cancer** | **Intervention evaluated** | **Study design** | **N studies** | **N patients** | **Patient-level correlation** | **Trial-level correlation between aggregated measures** | **Trial-level correlation between treatment effect** | **STE** | **Author’s conclusion** | **Surrogacy arguments** |
| --- | --- | --- | --- | --- | --- | --- | --- | --- | --- | --- | --- | --- |
| **Lung** | | | | | | | | | | | | |
| Laporte Silvy (2013) (39) | NSCLC | Advanced | Chemotherapy | IPD meta-analysis | 5 | 2331 | r= NR  R²= NR | r= NR  R²= NR | r= NR  R²= 0.62  [0.52;0.72] | 0.490 | Validation | R² ≥ 0.6 at trial level |
| Imai H (2014)(41) | NSCLC | Advanced | Chemotherapy | RCT |  | 39 | r= 0.76  R²= 0.5 | r= NR  R²= NR | r= NR  R²= NR | NR | Partial validation | No quantitative arguments |
| Imai H. (2014) (43) | NSCLC | Advanced | Chemotherapy | RCT |  | 50 | r= 0.67  R²= 0.39* | r= NR  R²= NR | r= NR  R²= NR | NR | Partial validation | No quantitative arguments |
| Pfeiffer B. (2017) (36)(34)(28) | NSCLC | Advanced | Chemotherapy | AD meta-analysis | 146 | 43061 | r= NR  R²= NR | r= 0.25  R²= NR | r= NR  R²= NR | 4.150^§^ | Partial validation | No quantitative arguments |
| Hotta K. (2011)(42) | NSCLC | Advanced | Chemotherapy/ targeted therapy | AD meta-analysis | 70 | 38721 | r= NR  R²=NR | r= NR  R²= 0.26* | r= NR  R²= NR | NR | No validation | No quantitative arguments |
| Hayashi H (2012) (45) | NSCLC | Advanced | Chemotherapy/ targeted therapy | AD meta-analysis | 69 | 37986 | r= NR  R²=NR | r= 0.43  R²= NR | r= NR  R²= NR | NR | Partial validation | No quantitative arguments |
| Petrelli F (2013)(44) | NSCLC | Advanced | Chemotherapy/ targeted therapy | AD meta-analysis | 12 | 4176 | r= NR  R²=NR | r= 0/37  R²<0.01* | r= 0.64  R²= NR | NR | No validation | No quantitative arguments |
| Hayashi H (2013) (43) | NSCLC | Advanced | Chemotherapy/ targeted therapy | AD meta-analysis | 18 | 11310 | r= NR  R²=NR | r= 0.51  R²= NR | r= 0.29  R²= NR | NR | No validation | No quantitative arguments |
| Aboshi M (2014)(17) | NSCLC | Advanced | Chemotherapy/ targeted therapy | AD meta-analysis | 65 | 23337 | r= NR  R²=NR | r= 0.689  R²= 0.439* | r= NR  R²= NR | NR | Partial validation | No quantitative arguments |
| Blumenthal G. (2015)(46) | NSCLC | Advanced | Chemotherapy/ targeted therapy | IPD meta-analysis | 15 | 12567 | r= NR  R²= NR | r= NR  R²= NR | r= NR  R²= 0.08  [0;0.31] | NR | No validation | No quantitative arguments |
| Nakashima K (2016)(47) | NSCLC | Advanced | Chemotherapy/ targeted therapy | AD meta-analysis | 44 | 22709 | r= NR  R²= NR | r= NR  R²= NR | r= 0.50  R²= 0.22* | NR | Validation | No quantitative arguments |
| Yoshino R (2014)(48) | NSCLC | Advanced | Immunotherapy/ chemotherapy/ targeted therapy | RCT |  | 35 | r= 0.13  R²= 0.001* | r= NR  R²= NR | r= NR  R²= NR | NR | No validation | No quantitative arguments |
| Zhao S (2018)(49) | NSCLC | Advanced | Immunotherapy/ chemotherapy/ targeted therapy | AD meta-analysis | 50 | 22804 | r= NR  R²= NR | r= NR  R²= 0.1* | r= NR  R²= 0.06  [0.06;0.07] | NR | No validation | No quantitative arguments |
| Li X (2012)(50) | NSCLC | Advanced | Targeted therapy | AD meta-analysis | 60 | 9903 | r= NR  R²=NR | r= NR  R²= 0.7* | r= NR  R²= NR | NR | Validation | R² ≥ 0.6 at trial level |
| Hotta K (2015)(51) | NSCLC | Advanced | Targeted therapy | AD meta-analysis | 18 | 7633 | r= NR  R²= NR | r= NR  R²= NR | r= NR  R²= 0.233 | NR | No validation | No quantitative arguments |
| Mauguen A (2013)(52) | NSCLC | Advanced/localized | Chemotherapy/ radiotherapy | IPD meta-analysis |  | 15071 | r= 0.77  R²=NR | r= NR  R²= NR | r= NR  R²= 0.96  [0.93;0.99] | 0.930 | Validation | R² ≥ 0.6 at trial level |
| Foster NR (2015)(53) | Extensive stage SCLC | Advanced | Chemotherapy | IPD meta-analysis | 7 | 2259 | r= NR  R²= NR | r= NR  R²= NR | r= NR  R²= 0.83  [0.43;0.95] | 0.670 | Validation | R² ≥ 0.6 at trial level |
| Imai H (2015)(54) | Extensive stage SCLC | Advanced | Chemotherapy | RCT |  | 49 | r= 0.58  R²= 0.24* | r= NR  R²= NR | r= NR  R²= NR | NR | No validation | No quantitative arguments |
| Nickolich M (2014)(55) | SCLC | Advanced | Chemotherapy/ targeted therapy | AD meta-analysis | 66 | 8471 | r= NR  R²=NR | r= 0.82  R²= NR | r= NR  R²= NR | NR | Validation | Some other arguments in favor of surrogacy |
| Wang X (2017)(56) | Mesothelioma | Advanced | Chemotherapy | IPD meta-analysis | 17 | 716 | r= NR  R²= 0.69* | r= NR  R²= NR | r= NR  R²= NR | NR | No validation | Some other arguments in favor of surrogacy |
| **Colorectal** | | | | | | | | | | | | |
| Buyse M (2007)(57) | Colorectal | Advanced | Chemotherapy | IPD meta-analysis | 10 | 3089 | r= 0.82  R²= NR | r= NR  R²= NR | r= NR  R²= 0.99  [0.84;1.04] | 0.860 | Validation | R² ≥ 0.6 at trial level |
| Tang PA (2007)(58) | Colorectal | Advanced | Chemotherapy | AD meta-analysis | 39 | 18668 | r= NR  R²= NR | r= 0.79  R²= NR | r= 0.74  R²= 0.65* | NR | Validation | R² ≥ 0.6 at trial level |
| Chibaudel B (2011)(59) | Colorectal | Advanced | Chemotherapy | IPD meta-analysis | 3 | 1042 | r= NR  R²= 0.56* | r= NR  R²= NR | r= NR  R²= 0.471  [0;1] | NR | No validation | No quantitative arguments |
| Giessen C (2015)(60) | Colorectal | Advanced | Chemotherapy | AD meta-analysis | 23 | 10800 | r= NR  R²= NR | r= 0.73  R²= NR | r= NR  R²= NR | NR | Validation | No quantitative arguments |
| Cicero G (2018)(61) | Colorectal | Advanced | Chemotherapy | RCT |  | 120 | r= 0.64  R²= 0.5* | r= NR  R²= NR | r= NR  R²= NR | NR | Validation | No quantitative arguments |
| Bonnetain F (2012)(62) | Colorectal | Advanced | Chemotherapy/ radiotherapy | IPD meta-analysis | 2 | 1767 | r= NR  R²= NR | r= NR  R²= NR | r= NR  R²= 0.88  [0.77;1] | NR | Validation | R² ≥ 0.6 at trial level |
| Giessen C (2012)(63) | Colorectal | Advanced | Chemotherapy/ targeted therapy | AD meta-analysis | 50 | 22736 | r= NR  R²= NR | r= NR  R²= 0.86* | r= NR  R²= 0.87  [0.67;0.93] | NR | Validation | R² ≥ 0.6 at trial level |
| Chirila C (2012)(64) | Colorectal | Advanced | Chemotherapy/ targeted therapy | AD meta-analysis | 66 | 23527 | r= NR  R²=NR | r= 0.89  R²= NR | r= NR  R²= 0.59* | NR | Validation | Some other arguments in favor of surrogacy |
| Shi Q (2015)(65) | Colorectal | Advanced | Chemotherapy/ targeted therapy | IPD meta-analysis | 22 | 16762 | r= 0.51  R²= NR | r= NR  R²= 0.69* | r= NR  R²= 0.54  [0.33;0.75] | 0.571 | Validation | R² ≥ 0.6 at trial level |
| Cremolini C (2017)(66) | Colorectal | Advanced | Chemotherapy/ targeted therapy | AD meta-analysis | 20 | 7571 | r= NR  R²= NR | r= NR  R²= NR | r= 0.734  R²= 0.54* | NR | No validation | No quantitative arguments |
| Sidhu R (2013) (67) | Colorectal | Advanced | Immunotherapy/ chemotherapy/ targeted therapy | AD meta-analysis | 24 | 20438 | r= NR  R²= NR | r= NR  R²= NR | r= 0.86  R²= 0.73  [0.53;0.85] | 0.900 | Validation | R² ≥ 0.6 at trial level |
| Petrelli F (2013)(68) | Colorectal | Advanced | Immunotherapy/ chemotherapy/ targeted therapy | AD meta-analysis | 34 | 16408 | r= NR  R²= NR | r= 0.64  R²= 0.43* | r= 0.59  R²= NR | NR | Partial validation | No quantitative arguments |
| Ciani O (2015)(69) | Colorectal | Advanced | Immunotherapy/ chemotherapy/ targeted therapy | AD meta-analysis | 101 | 40243 | r= NR  R²= NR | r= NR  R²= NR | r= 0.75  R²= 0.34  [0.1;0.59] | 0.800 | No validation | No quantitative arguments |
| Montagnani F. (2016)(70) | Colorectal | Advanced | Targeted therapy | AD meta-analysis | 13 | 7389 | r= NR  R²= NR | r= NR  R²= NR | r= NR  R²= 0.68* | 0.890 | Validation | R² ≥ 0.6 at trial level |
| Colloca G. (2016)(71) | Colorectal | Advanced | Targeted therapy | AD meta-analysis | 11 |  | r= NR  R²= NR | r= 0.817  R²= NR | r= NR  R²= 0.71* | NR | Validation | R² ≥ 0.6 at trial level |
| **Breast cancer** | | | | | | | | | | | | |
| Hackshaw A (2005)(72) | Breast | Advanced | Chemotherapy | AD meta-analysis | 42 | 9163 | r= NR  R²= NR | r= NR  R²= NR | r= NR  R²= 0.56* | NR | Validation | No quantitative arguments |
| Burzykowski T (2008)(73) | Breast | Advanced | Chemotherapy | IPD meta-analysis | 11 | 3953 | r= 0.69  R²= NR | r= NR  R²= NR | r= 0.48  R²= NR | NR | No validation | No quantitative arguments |
| Miksad R. (2008)(74) | Breast | Advanced | Chemotherapy | AD meta-analysis | 16 | 4323 | r= NR  R²= NR | r= 0.71  R²= NR | r= NR  R²= 0.49* | NR | Validation | No quantitative arguments |
| Culakova E. (2016)(75) | Breast | Advanced | Chemotherapy | AD meta-analysis | 70 | 15043 | r= NR  R²= NR | r= NR  R²= NR | r= 0.49  R²= NR | NR | No validation | No quantitative arguments |
| Kundu MG (2017)(76,77) | Breast | Advanced | Chemotherapy | AD meta-analysis | 65 |  | r= NR  R²= NR | r= NR  R²= NR | r= NR  R²= NR | 0.448 | Validation | No quantitative arguments |
| Sherrill B (2008)(78) | Breast | Advanced | Chemotherapy/ targeted therapy | AD meta-analysis | 67 | 17079 | r= NR  R²= NR | r= 0.38  R²= NR | r= NR  R²= 0.3* | NR | No validation | No quantitative arguments |
| Beauchemin C (2014)(79) | Breast | Advanced | Chemotherapy/ targeted therapy | AD meta-analysis | 144 | 43459 | r= NR  R²= NR | r= 0.43  R²= NR | r= 0.43  R²= 0.86* | NR | Validation | R² ≥ 0.6 at trial level |
| Petrelli F. (2014)(38) | Breast | Advanced | Chemotherapy/ targeted therapy | AD meta-analysis | 20 | 10138 | r= NR  R²= NR | r= 0.81  R²= 0.61* | r= 0.7  R²= 0.73* | NR | Partial validation | R² ≥ 0.6 at trial level |
| Adunlin G (2015)(18) | Breast | Advanced | Chemotherapy/ targeted therapy | AD meta-analysis | 72 |  | r= NR  R²= NR | r= NR  R²= NR | r= 0.46  R²= 0.31* | NR | Partial validation | No quantitative arguments |
| Liu L. (2016)(80) | Breast | Advanced | Chemotherapy/ targeted therapy | AD meta-analysis | 24 | 8617 | r= NR  R²= NR | r= 0.78  R²= NR | r= NR  R²= 0.64* | NR | Partial validation | R² ≥ 0.6 at trial level |
| Li L. (2018) (81) | Breast | Advanced | Chemotherapy/ targeted therapy | AD meta-analysis | 37 | 14966 | r= NR  R²= NR | r= 0.41  R²= NR | r= 0.56  R²= NR | NR | Validation | No quantitative arguments |
| Forsythe A (2018)(82) | Breast | Advanced | Chemotherapy/ targeted therapy | AD meta-analysis | 40 | 20000 | r= NR  R²= NR | r= 0.65  R²= NR | r= NR  R²= 0.35* | 1.130 | Validation | No quantitative arguments |
| Matsubara Y (2011)(83) | Breast | Advanced/localized | Chemotherapy | RCT |  | 30 | r= 0.99  R²= NR | r= NR  R²= NR | r= NR  R²= NR | NR | No validation | Some other arguments in favor of surrogacy |
| **Hematological** | | | | | | | | | | | | |
| Shi Q (2018)(84) | Hematological | Advanced | Chemotherapy/ targeted therapy | IPD meta-analysis | 13 | 7507 | r= 0.85  R²= NR | r= NR  R²= NR | r= NR  R²= 0.83  [0.57;0.94] | 0.890 | Validation | R² ≥ 0.6 at trial level |
| Zhu R (2017)(85) | Hematological | Advanced | Immunotherapy/ chemotherapy/ targeted therapy | AD meta-analysis | 108 | 10036 | r= NR  R²= NR | r= NR  R²= 0.81* | r= NR  R²= NR | NR | Validation | R² ≥ 0.6 at trial level |
| Felix J (2013)(86) | Hematological | Advanced/localized | Chemotherapy | AD meta-analysis | 153 | 22696 | r= NR  R²= NR | r= 0.75  R²= NR | r= NR  R²= NR | NR | Partial validation | No quantitative arguments |
| Jiménez-Ubieto A (2017)(87) | Hematological | Advanced/localized | Chemotherapy/ surgery | RCT |  |  | r= NR  R²= NR | r= NR  R²= NR | r= NR  R²= NR | NR | Validation | No quantitative arguments |
| Cartier S (2015)(88) | Hematological | Advanced/localized | Chemotherapy/ targeted therapy | AD meta-analysis | 21 | 12048 | r= NR  R²= NR | r= NR  R²= NR | r= 0.82  R²= 0.63  [0.43;0.84] | NR | Validation | R² ≥ 0.6 at trial level |
| Beauchemin C (2015)(89) | Hematological | Advanced/localized | Immunotherapy/ chemotherapy/ targeted therapy | AD meta-analysis | 23 |  | r= NR  R²= NR | r= 0.81  R²= NR | r= NR  R²= NR | NR | Validation | Some other arguments in favor of surrogacy |
| **Gastric** | | | | | | | | | | | | |
| Paoletti X (2013)(90) | Gastric | Advanced | Chemotherapy | IPD meta-analysis | 20 | 4069 | r= 0.85  R²= NR | r= NR  R²= NR | r= NR  R²= 0.61  [0.04. 1.00] | 0.560 | No validation | R² ≥ 0.6 at trial level |
| Shitara K (2014)(19) | Gastric | Advanced | Chemotherapy | AD meta-analysis | 64 | 4286 | r= NR  R²= NR | r= 0.56  R²= NR | r= 0.36  R²= NR | NR | No validation | No quantitative arguments |
| Liu L (2015)(91) | Gastric | Advanced | Chemotherapy | IPD meta-analysis | 2 | 303 | r= 0.84  R²= NR | r= NR  R²= NR | r= NR  R²= NR | NR | Validation | Some other arguments in favor of surrogacy |
| Ozer-Stillman I (2014)(92) | Gastric | Advanced | Chemotherapy/ targeted therapy | AD meta-analysis | 19 | 2189 | r= NR  R²= NR | r= 0.91  R²= 0.84* | r= NR  R²= NR | NR | Validation | R² ≥ 0.6 at trial level |
| Shitara K (2012) (93) | Gastric | Advanced | Immunotherapy/ chemotherapy | AD meta-analysis | 36 | 10484 | r= NR  R²= NR | r= 0.7  R²= NR | r= 0.8  R²= NR | NR | Validation | Some other arguments in favor of surrogacy |
| **Head and neck** | | | | | | | | | | | | |
| Chen YP (2015)(94) | Head and neck | Advanced | Chemotherapy | RCT |  | 208 | r= 0.73  R²= NR | r= NR  R²= NR | r= NR  R²= NR | NR | Validation | No quantitative arguments |
| Rotolo F (2017)(15) | Head and neck | Advanced | Chemotherapy | IPD meta-analysis | 20 | 5360 | r= 0.93  R²= NR | r= NR  R²= NR | r= NR  R²= 0.95  [0.47;1] | 0.890 | Validation | R² ≥ 0.6 at trial level |
| Chen Y (2015)(95) | Head and neck | Advanced/localized | Chemotherapy/ radiotherapy | AD meta-analysis | 21 | 5212 | r= NR  R²= NR | r= NR  R²= NR | r= NR  R²= 0.9* | 0.880 | Validation | R² ≥ 0.6 at trial level |
| Kataoka K (2017)(96) | Head and neck | Localized | Chemotherapy/ radiotherapy | AD meta-analysis | 10 | 2851 | r= NR  R²= NR | r= NR  R²= NR | r= NR  R²= 0.28  [0.0/ 0.9] | NR | No validation | No quantitative arguments |
| **Pancreas** | | | | | | | | | | | | |
| Petrelli F (2015)(97) | Pancreas | Advanced | Chemotherapy | AD meta-analysis | 30 | 8467 | r= NR  R²=NR | r= 0.75  R²= 0.6* | r= 0.78  R²= 0.69* | NR | Validation | R² ≥ 0.6 at trial level |
| Makris E/ (2017)(98) | Pancreas | Advanced | Chemotherapy | AD meta-analysis | 24 | 10708 | r= NR  R²= NR | r= NR  R²= NR | r= 0.86  R²= NR | NR | Validation | Some other arguments in favor of surrogacy |
| Hamada T (2016)(99) | Pancreas | Advanced | Chemotherapy/ targeted therapy | AD meta-analysis | 50 | 15906 | r= NR  R²= NR | r= 0.76  R²= NR | r= 0.75  R²= 0.84  [0.62;0.89] | NR | Validation | R² ≥ 0.6 at trial level |
| Colloca G (2016)(100) | Pancreas | Advanced | Chemotherapy/ targeted therapy | AD meta-analysis | 37 |  | r= NR  R²= NR | r= NR  R²= NR | r= 0.67  R²= NR | NR | Validation | No quantitative arguments |
| Dabbous O (2017)(101) | Pancreas | Advanced | Immunotherapy/ chemotherapy/ targeted therapy | AD meta-analysis | 28 | 6734 | r= NR  R²=NR | r= 0.84  R²= NR | r= NR  R²= NR | NR | Validation | Some other arguments in favor of surrogacy |
| **Many localizations** | | | | | | | | | | | | |
| Wilkerson J (2009)(102) | Breast/gastric/ colorectal/ ovarian/ pancreas/renal | Advanced/localized | Chemotherapy | AD meta-analysis | 66 |  | r= NR  R²=NR | r= NR  R²= NR | r= NR  R²= 0.62* | NR | No validation | R² ≥ 0.6 at trial level |
| Amir E (2012)(103) | Breast/head and neck/ lung/HCC/ colorectal/ hematological/ pancreas/renal | Advanced | Chemotherapy/ targeted therapy | AD meta-analysis | 26 |  | r= NR  R²=NR | r= NR  R²= NR | r= 0.64  R²= NR | NR | No validation | No quantitative arguments |
| Kaufman H (2017)(104) | Many localizations | Advanced/localized | Immunotherapy | AD meta-analysis | 18 | 7140 | r= NR  R²=NR | r= NR  R²= NR | r= NR  R²= 0* | NR | No validation | No quantitative arguments |
| Tan A (2017)(105) | Breast/head and neck/central nervous system/ lung/HCC/ ovarian/ gynecological/ pancreas | Advanced/localized | Immunotherapy | AD meta-analysis | 51 |  | r= NR  R²=NR | r= NR  R²= NR | r= 0.62  R²= 0.38  [0.15;0.58] | 0.500 | No validation | No quantitative arguments |
| Mushti SL (2018)(106) | Head and neck/ melanoma/ lung/renal | Advanced/localized | Immunotherapy | IPD meta-analysis | 13 | 6722 | r= 0.61  R²=NR | r= NR  R²= NR | r= NR  R²= 0.13* | NR | No validation | No quantitative arguments |
| Nie R (2019)(107) | Solid tumors | Advanced | Immunotherapy | AD meta-analysis | 43 | 15088 | r= NR  R²=NR | r= 0.54  R²= NR | r= NR  R²= 0.37* | 1.070 | No validation | No quantitative arguments |
| **Renal** | | | | | | | | | | | | |
| Halabi S (2014)(108) | Renal | Advanced | Chemotherapy/ targeted therapy | IPD meta-analysis | 2 | 1381 | r= NR  R²= 0.53* | r= NR  R²= NR | r= NR  R²= NR | NR | Validation | No quantitative arguments |
| Delea TE (2012)(109) | Renal | Advanced | Immunotherapy/ chemotherapy/ targeted therapy | AD meta-analysis | 31 | 10943 | r= NR  R²= NR | r= NR  R²= NR | r= 0.8  R²= 0.63* | NR | Validation | R² ≥ 0.6 at trial level |
| Johnson KR (2015)(110) | Renal | Advanced | Immunotherapy/ chemotherapy/ targeted therapy | AD meta-analysis | 30 |  | r= NR  R²= NR | r= NR  R²= NR | r= 0.54  R²= 0.49* | 3.650 | Partial validation | No quantitative arguments |
| Petrelli F (2013)(111) | Renal | Advanced/localized | Chemotherapy/ targeted therapy | AD meta-analysis | 10 | 3188 | r= NR  R²= NR | r= 0.87  R²= 0.97* | r= 0.36  R²= 0.07* | NR | No validation | No quantitative arguments |
| **Soft tissue sarcoma** | | | | | | | | | | | | |
| Savina M (2018)(112) | Soft tissue sarcoma | Advanced | Chemotherapy/ targeted therapy | IPD meta-analysis | 14 | 2846 | r= 0.62  R²=NR | r= NR  R²= NR | r= NR  R²= 0.33  [0;0.6] | NR | No validation | No quantitative arguments |
| Zer A (2016)(113) | Soft tissue sarcoma | Advanced | Immunotherapy/ chemotherapy/ targeted therapy | AD meta-analysis | 52 | 9762 | r= NR  R²=NR | r= NR  R²= NR | r= 0.61  R²= NR | NR | Validation | No quantitative arguments |
| Tanaka K (2019)(20) | Soft tissue sarcoma | Advanced | Immunotherapy/ chemotherapy/ targeted therapy | AD meta-analysis | 27 | 6156 | r= NR  R²=NR | r= NR  R²= NR | r= NR  R²= 0.56  [0.33;0.79] | NR | No validation | No quantitative arguments |
| **Melanoma** | | | | | | | | | | | | |
| Flaherty KT (2014)(114) | Melanoma | Advanced | Chemotherapy/ targeted therapy | AD meta-analysis | 12 | 4416 | r= NR  R²= NR | r= NR  R²= NR | r= 0.89  R²= NR | NR | Validation | Some other arguments in favor of surrogacy |
| Petrelli F (2016)(115) | Melanoma | Advanced | Immunotherapy | AD meta-analysis | 13 | 3373 | r= NR  R²=NR | r= 0.45  R²= 0.21* | r= NR  R²= NR | NR | Partial validation | No quantitative arguments |
| **Ovarian** | | | | | | | | | | | | |
| Sjoquist KM (2018)(116) | Ovarian | Advanced | Chemotherapy | AD meta-analysis | 26 | 24870 | r= NR  R²= NR | r= NR  R²= NR | r= NR  R²= 0.52  [0.3;0.67] | NR | Partial validation | No quantitative arguments |
| Colloca G (2017)(117) | Ovarian | Advanced | Chemotherapy/ targeted therapy | AD meta-analysis | 38 |  | r= NR  R²=NR | r= NR  R²= NR | r= 0.55  R²= NR | NR | No validation | No quantitative arguments |
| **Biliary** | | | | | | | | | | | | |
| Moriwaki T (2016)(118) | Billiary | Advanced | Chemotherapy/ targeted therapy | AD meta-analysis | 17 | 2148 | r= NR  R²= NR | r= NR  R²= NR | r= NR  R²= 0.66  [0.32;0.85] | 0.830 | Validation | R² ≥ 0.6 at trial level |
| **Central nervous system** | | | | | | | | | | | | |
| Han K (2014)(119) | Glioblastoma | Advanced/localized | Chemotherapy/ targeted therapy | AD meta-analysis | 91 | 7125 | r= NR  R²= NR | r= NR  R²= 0.7 | r= NR  R²= 0.92  [0.71;0.99] | NR | Validation | R² ≥ 0.6 at trial level |
| Imaoka H (2017)(120) | euro-endocrine tumors | Advanced | Chemotherapy/ targeted therapy | AD meta-analysis | 23 | 2530 | r= NR  R²=NR | r= 0.59  R²= NR | r= 0.17  R²= NR | NR | Validation | No quantitative arguments |
| **Urothelial** | | | | | | | | | | | | |
| Liang F (2018)(121) | Urothelial | Advanced | Chemotherapy. targeted therapy | AD meta-analysis | 48 | 7019 | r= NR  R²=NR | r= NR  R²= NR | r= NR  R²= 0.79  [0.58;0.91] | NR | Partial validation | R² ≥ 0.6 at trial level |
| **Gynecological** | | | | | | | | | | | | |
| Rose PG (2010)(122) | Gynecological | Advanced | Chemotherapy | IPD meta-analysis | 11 | 407 | r= 0.661  R²=NR | r= NR  R²= NR  $Ʈ_{Kendall}=0.514$ | r= NR  R²= NR | NR | Validation | No quantitative arguments |
| **Urinary carcinoma** | | | | | | | | | | | | |
| Abdel-Rahman O (2018)(123) | Urinary carcinoma | Advanced | Immunotherapy | AD meta-analysis | 13 | 1699 | r= NR  R²=NR | r= -0.02  R²= NR | r= NR  R²= NR | NR | No validation | No quantitative arguments |

* 95% confidence interval was not reported

§ Median progression-free survival

NR: not reported

HCC, hepatocellular carcinoma; NSCLC, non-small-cell lung cancer; SCLC, small-cell lung cancer; AD, all data; RCT, randomized controlled trial; IPD, individual patient data

Reference lists :

36. Pfeiffer B, Hashim M, Bartsch R, Postma M, Heeg B. Objective response rate and progression-free survival as surrogate endpoints for overall survival and the impact of crossover and unbalanced post-progression treatments: a systematic review and meta-analysis in second- and furtherline therapy of advanced non-small cell lung cancer. J Clin Oncol Conf 2017 Annu Meet Am Soc Clin Oncol ASCO U S. 2017;35(15 Supplement 1) (no pagination).

37. Broglio KR, Berry DA. Detecting an overall survival benefit that is derived from progression-free survival. J Natl Cancer Inst. 2009 Dec 2;101(23):1642–9.

38. Petrelli F, Barni S. Surrogate endpoints in metastatic breast cancer treated with targeted therapies: an analysis of the first-line phase III trials. Med Oncol Northwood Lond Engl. 2014 Jan;31(1):776.

39. Laporte S, Squifflet P, Baroux N, Fossella F, Georgoulias V, Pujol J-L, et al. Prediction of survival benefits from progression-free survival benefits in advanced non-small-cell lung cancer: evidence from a meta-analysis of 2334 patients from 5 randomised trials. BMJ Open. 2013 Mar 13;3(3).

40. Imai H, Mori K, Ono A, Akamatsu H, Taira T, Kenmotsu H, et al. Individual-level data on the relationships of progression-free survival and post-progression survival with overall survival in patients with advanced non-squamous non-small cell lung cancer patients who received second-line chemotherapy. Med Oncol Northwood Lond Engl. 2014 Aug;31(8):88.

41. Imai H, Takahashi T, Mori K, Ono A, Akamatsu H, Shukuya T, et al. Individual-level data on the relationships of progression-free survival, post-progression survival, and tumor response with overall survival in patients with advanced non-squamous non-small cell lung cancer. Neoplasma. 2014;61(2):233–40.

42. Hotta K, Kiura K, Fujiwara Y, Takigawa N, Hisamoto A, Ichihara E, et al. Role of Survival Post-Progression in Phase III Trials of Systemic Chemotherapy in Advanced Non-Small-Cell Lung Cancer: A Systematic Review. PLOS ONE. 2011 Nov 17;6(11):e26646.

43. Hayashi H, Okamoto I, Morita S, Taguri M, Nakagawa K. Postprogression survival for first-line chemotherapy of patients with advanced non-small-cell lung cancer. Ann Oncol. 2012 Jun 1;23(6):1537–41.

44. Petrelli F, Barni S. Is overall survival still the primary endpoint in maintenance non-small cell lung cancer studies? An analysis of phase III randomised trials. Transl Lung Cancer Res. 2013 Feb;2(1):6–13.

45. XXREF Hayashi 2012

46. Blumenthal GM, Karuri SW, Zhang H, Zhang L, Khozin S, Kazandjian D, et al. Overall response rate, progression-free survival, and overall survival with targeted and standard therapies in advanced non-small-cell lung cancer: US Food and Drug Administration trial-level and patient-level analyses. J Clin Oncol Off J Am Soc Clin Oncol. 2015 Mar 20;33(9):1008–14.

47. Nakashima K, Horita N, Nagai K, Manabe S, Murakami S, Ota E, et al. Progression-free survival, response rate, and disease control rate as predictors of overall survival in phase III Randomized controlled trials evaluating the first-line chemotherapy for advanced, locally advanced, and recurrent non-small cell lung Carcinoma. J Thorac Oncol. 2016;11(9):1574–85.

48. Yoshino R, Imai H, Mori K, Takei K, Tomizawa M, Kaira K, et al. Surrogate endpoints for overall survival in advanced non-small-cell lung cancer patients with mutations of the epidermal growth factor receptor gene. Mol Clin Oncol. 2014 Sep;2(5):731–6.

49. Zhao S, Zhang Z, Zhang Y, Hong S, Zhou T, Yang Y, et al. Progression-free survival and one-year milestone survival as surrogates for overall survival in previously treated advanced non-small cell lung cancer. Int J Cancer. 2018 Nov 14;

50. Li X, Liu S, Gu H, Wang D. Surrogate end points for survival in the target treatment of advanced non-small-cell lung cancer with gefitinib or erlotinib. J Cancer Res Clin Oncol. 2012 Nov 1;138(11):1963–9.

51. Hotta K, Kato Y, Leighl N, Takigawa N, Gaafar RM, Kayatani H, et al. Magnitude of the benefit of progression-free survival as a potential surrogate marker in phase 3 trials assessing targeted agents in molecularly selected patients with advanced non-small cell lung cancer: systematic review. PloS One. 2015;10(3):e0121211.

52. Mauguen A, Pignon J-P, Burdett S, Domerg C, Fisher D, Paulus R, et al. Surrogate endpoints for overall survival in chemotherapy and radiotherapy trials in operable and locally advanced lung cancer: a re-analysis of meta-analyses of individual patients’ data. Lancet Oncol. 2013 Jun;14(7):619–26.

53. Foster NR, Qi Y, Shi Q, Krook JE, Kugler JW, Jett JR, et al. Tumor response and progression-free survival as potential surrogate endpoints for overall survival in extensive stage small-cell lung cancer: findings on the basis of North Central Cancer Treatment Group trials. Cancer. 2011 Mar 15;117(6):1262–71.

54. Imai H, Mori K, Wakuda K, Ono A, Akamatsu H, Shukuya T, et al. Progression-free survival, post-progression survival, and tumor response as surrogate markers for overall survival in patients with extensive small cell lung cancer. Ann Thorac Med. 2015 Mar;10(1):61–6.

55. Nickolich M, Babakoohi S, Fu P, Dowlati A. Clinical trial design in small cell lung cancer: surrogate end points and statistical evolution. Clin Lung Cancer. 2014 May;15(3):207–12.

56. Wang X, Wang X, Hodgson L, George SL, Sargent DJ, Foster NR, et al. Validation of Progression-Free Survival as a Surrogate Endpoint for Overall Survival in Malignant Mesothelioma: Analysis of Cancer and Leukemia Group B and North Central Cancer Treatment Group (Alliance) Trials. The Oncologist. 2017 Feb;22(2):189–98.

57. Buyse M, Burzykowski T, Carroll K, Michiels S, Sargent DJ, Miller LL, et al. Progression-free survival is a surrogate for survival in advanced colorectal cancer. J Clin Oncol Off J Am Soc Clin Oncol. 2007 Nov 20;25(33):5218–24.

58. Tang PA, Bentzen SM, Chen EX, Siu LL. Surrogate end points for median overall survival in metastatic colorectal cancer: literature-based analysis from 39 randomized controlled trials of first-line chemotherapy. J Clin Oncol Off J Am Soc Clin Oncol. 2007 Oct 10;25(29):4562–8.

59. Chibaudel B, Bonnetain F, Shi Q, Buyse M, Tournigand C, Sargent DJ, et al. Alternative end points to evaluate a therapeutic strategy in advanced colorectal cancer: evaluation of progression-free survival, duration of disease control, and time to failure of strategy--an Aide et Recherche en Cancerologie Digestive Group Study. J Clin Oncol Off J Am Soc Clin Oncol. 2011 Nov 1;29(31):4199–204.

60. Giessen C, Laubender RP, Ankerst DP, Stintzing S, Modest DP, Schulz C, et al. Surrogate endpoints in second-line treatment for mCRC: a systematic literature-based analysis from 23 randomised trials. Acta Oncol Stockh Swed. 2015 Feb;54(2):187–93.

61. Cicero G, De Luca R, Dieli F. Progression-free survival as a surrogate endpoint of overall survival in patients with metastatic colorectal cancer. OncoTargets Ther. 2018;11:3059–63.

62. Bonnetain F, Bosset JF, Gerard JP, Calais G, Conroy T, Mineur L, et al. What is the clinical benefit of preoperative chemoradiotherapy with 5FU/leucovorin for T3-4 rectal cancer in a pooled analysis of EORTC 22921 and FFCD 9203 trials: surrogacy in question? Eur J Cancer Oxf Engl 1990. 2012 Aug;48(12):1781–90.

63. Giessen C, Laubender RP, Ankerst DP, Stintzing S, Modest DP, Mansmann U, et al. Progression-free survival as a surrogate endpoint for median overall survival in metastatic colorectal cancer: literature-based analysis from 50 randomized first-line trials. Clin Cancer Res Off J Am Assoc Cancer Res. 2013 Jan 1;19(1):225–35.

64. Chirila C, Odom D, Devercelli G, Khan S, Sherif BN, Kaye JA, et al. Meta-analysis of the association between progression-free survival and overall survival in metastatic colorectal cancer. Int J Colorectal Dis. 2012 May;27(5):623–34.

65. Shi Q, de Gramont A, Grothey A, Zalcberg J, Chibaudel B, Schmoll H-J, et al. Individual patient data analysis of progression-free survival versus overall survival as a first-line end point for metastatic colorectal cancer in modern randomized trials: findings from the analysis and research in cancers of the digestive system database. J Clin Oncol Off J Am Soc Clin Oncol. 2015 Jan 1;33(1):22–8.

66. Cremolini C, Antoniotti C, Pietrantonio F, Berenato R, Tampellini M, Baratelli C, et al. Surrogate Endpoints in Second-Line Trials of Targeted Agents in Metastatic Colorectal Cancer: A Literature-Based Systematic Review and Meta-Analysis. Cancer Res Treat Off J Korean Cancer Assoc. 2017;49(3):834–45.

67. Sidhu R, Rong A, Dahlberg S. Evaluation of progression-free survival as a surrogate endpoint for survival in chemotherapy and targeted agent metastatic colorectal cancer trials. Clin Cancer Res Off J Am Assoc Cancer Res. 2013 Mar 1;19(5):969–76.

68. Petrelli F, Barni S. Correlation of progression-free and post-progression survival with overall survival in advanced colorectal cancer. Ann Oncol Off J Eur Soc Med Oncol. 2013 Jan;24(1):186–92.

69. Ciani O, Buyse M, Garside R, Peters J, Saad ED, Stein K, et al. Meta-analyses of randomized controlled trials show suboptimal validity of surrogate outcomes for overall survival in advanced colorectal cancer. J Clin Epidemiol. 2015 Jul;68(7):833–42.

70. Montagnani F, DI Leonardo G, Pino MS, Martella F, Perboni S, Ribecco A, et al. Progression-free Survival as a Surrogate End-point in Advanced Colorectal Cancer Treated with Antiangiogenic Therapies. Anticancer Res. 2016 Aug;36(8):4259–65.

71. Colloca G, Venturino A, Guarneri D. Analysis of Clinical End Points of Randomised Trials Including Bevacizumab and Chemotherapy versus Chemotherapy as First-line Treatment of Metastatic Colorectal Cancer. Clin Oncol R Coll Radiol G B. 2016 Oct;28(10):e155-164.

72. Hackshaw A, Knight A, Barrett-Lee P, Leonard R. Surrogate markers and survival in women receiving first-line combination anthracycline chemotherapy for advanced breast cancer. Br J Cancer. 2005 Nov;93(11):1215–21.

73. Burzykowski T, Buyse M, Piccart-Gebhart MJ, Sledge G, Carmichael J, Lück H-J, et al. Evaluation of tumor response, disease control, progression-free survival, and time to progression as potential surrogate end points in metastatic breast cancer. J Clin Oncol Off J Am Soc Clin Oncol. 2008 Apr 20;26(12):1987–92.

74. Miksad RA, Zietemann V, Gothe R, Schwarzer R, Conrads-Frank A, Schnell-Inderst P, et al. Progression-free survival as a surrogate endpoint in advanced breast cancer. Int J Technol Assess Health Care. 2008;24(4):371–83.

75. Culakova E, Poniewierski M, Crawford J, Dale D, Lyman G. Relationship between overall survival and surrogate measures in patients with metastatic breast cancer treated with chemotherapy. Cancer Res Conf 39th Annu CTRC-AACR San Antonio Breast Cancer Symp U S. 2017;77(4 Supplement 1) (no pagination).

76. Kundu MG, Acharyya S. Surrogacy of progression free survival for overall survival in metastatic breast cancer studies: Meta-analyses of published studies. Contemp Clin Trials. 2017 Feb;53:20–8.

77. Kundu MG, Acharyya S. Corrigendum to “Surrogacy of progression free survival for overall survival in metastatic breast cancer studies: Meta-analyses of published studies” [Contemp. Clin. Trials 53 (2017) 20-28]. Contemp Clin Trials. 2017 Sep;60:126.

78. Sherrill B, Amonkar M, Wu Y, Hirst C, Stein S, Walker M, et al. Relationship between effects on time-to-disease progression and overall survival in studies of metastatic breast cancer. Br J Cancer. 2008 Nov;99(10):1572–8.

79. Beauchemin C, Cooper D, Lapierre M-È, Yelle L, Lachaine J. Progression-free survival as a potential surrogate for overall survival in metastatic breast cancer. OncoTargets Ther. 2014;7:1101–10.

80. Liu L, Chen F, Zhao J, Yu H. Correlation between overall survival and other endpoints in metastatic breast cancer with second- or third-line chemotherapy: literature-based analysis of 24 randomized trials. Bull Cancer (Paris). 2016;103(4):336–44.

81. Li L, Pan Z. Progression-Free Survival and Time to Progression as Real Surrogate End Points for Overall Survival in Advanced Breast Cancer: A Meta-Analysis of 37 Trials. Clin Breast Cancer. 2017 Jul 25;

82. Forsythe A, Chandiwana D, Barth J, Thabane M, Baeck J, Tremblay G. Progression-free survival/time to progression as a potential surrogate for overall survival in HR+, HER2- metastatic breast cancer. Breast Cancer Dove Med Press. 2018;10:69–78.

83. Matsubara Y, Sakabayashi S, Nishimura T, Ishida T, Ohuchi N, Teramukai S, et al. Surrogacy of tumor response and progression-free survival for overall survival in metastatic breast cancer resistant to both anthracyclines and taxanes. Int J Clin Oncol. 2011 Dec;16(6):623–9.

84. Shi Q, Schmitz N, Ou F-S, Dixon JG, Cunningham D, Pfreundschuh M, et al. Progression-Free Survival as a Surrogate End Point for Overall Survival in First-Line Diffuse Large B-Cell Lymphoma: An Individual Patient-Level Analysis of Multiple Randomized Trials (SEAL). J Clin Oncol Off J Am Soc Clin Oncol. 2018 Sep 1;36(25):2593–602.

85. Zhu R, Lu D, Chu Y-W, Chai A, Green M, Zhang N, et al. Assessment of Correlation Between Early and Late Efficacy Endpoints to Identify Potential Surrogacy Relationships in Non-Hodgkin Lymphoma: a Literature-Based Meta-analysis of 108 Phase II and Phase III Studies. AAPS J. 2017 May;19(3):669–81.

86. Félix J, Aragão F, Almeida JM, Calado FJ, Ferreira D, Parreira ABS, et al. Time-dependent endpoints as predictors of overall survival in multiple myeloma. BMC Cancer. 2013 Mar 16;13:122.

87. Jiménez-Ubieto A, Grande C, Caballero D, Yáñez L, Novelli S, Hernández MT, et al. Progression-free survival at 2 years post-autologous transplant: a surrogate end point for overall survival in follicular lymphoma. Cancer Med. 2017 Oct 26;

88. Cartier S, Zhang B, Rosen VM, Zarotsky V, Bartlett JB, Mukhopadhyay P, et al. Relationship between treatment effects on progression-free survival and overall survival in multiple myeloma: a systematic review and meta-analysis of published clinical trial data. Oncol Res Treat. 2015;38(3):88–94.

89. Beauchemin C, Johnston JB, Lapierre MÈ, Aissa F, Lachaine J. Relationship between progression-free survival and overall survival in chronic lymphocytic leukemia: a literature-based analysis. Curr Oncol Tor Ont. 2015 Jun;22(3):e148-156.

90. Paoletti X, Oba K, Bang Y-J, Bleiberg H, Boku N, Bouché O, et al. Progression-free survival as a surrogate for overall survival in advanced/recurrent gastric cancer trials: a meta-analysis. J Natl Cancer Inst. 2013 Nov 6;105(21):1667–70.

91. Liu L, Yu H, Huang L, Shao F, Bai J, Lou D, et al. Progression-free survival as a surrogate endpoint for overall survival in patients with third-line or later-line chemotherapy for advanced gastric cancer. OncoTargets Ther. 2015;8:921–8.

92. Özer-Stillman I, Strand L, Chang J, Mohamed AF, Tranbarger-Freier KE. Meta-analysis for the association between overall survival and progression-free survival in gastrointestinal stromal tumor. Clin Cancer Res Off J Am Assoc Cancer Res. 2015 Jan 15;21(2):295–302.

93. Shitara K, Ikeda J, Yokota T, Takahari D, Ura T, Muro K, et al. Progression-free survival and time to progression as surrogate markers of overall survival in patients with advanced gastric cancer: analysis of 36 randomized trials. Invest New Drugs. 2012 Jun;30(3):1224–31.

94. Chen Y-P, Zhang W-N, Tang L-L, Mao Y-P, Liu X, Chen L, et al. Identification of surrogate endpoints in patients with locoregionally advanced nasopharyngeal carcinoma receiving neoadjuvant chemotherapy plus concurrent chemoradiotherapy versus concurrent chemoradiotherapy alone. BMC Cancer. 2015 Nov 24;15:930.

95. Chen Y-P, Sun Y, Chen L, Mao Y-P, Tang L-L, Li W-F, et al. Surrogate endpoints for overall survival in combined chemotherapy and radiotherapy trials in nasopharyngeal carcinoma: Meta-analysis of randomised controlled trials. Radiother Oncol J Eur Soc Ther Radiol Oncol. 2015 Aug;116(2):157–66.

96. Kataoka K, Nakamura K, Mizusawa J, Kato K, Eba J, Katayama H, et al. Surrogacy of progression-free survival (PFS) for overall survival (OS) in esophageal cancer trials with preoperative therapy: Literature-based meta-analysis. Eur J Surg Oncol J Eur Soc Surg Oncol Br Assoc Surg Oncol. 2017 Oct;43(10):1956–61.

97. Petrelli F, Coinu A, Borgonovo K, Cabiddu M, Barni S. Progression-free survival as surrogate endpoint in advanced pancreatic cancer: meta-analysis of 30 randomized first-line trials. Hepatobiliary Pancreat Dis Int HBPD INT. 2015 Apr;14(2):124–31.

98. Makris EA, MacBarb R, Harvey DJ, Poultsides GA. Surrogate End Points for Overall Survival in Metastatic, Locally Advanced, or Unresectable Pancreatic Cancer: A Systematic Review and Meta-Analysis of 24 Randomized Controlled Trials. Ann Surg Oncol. 2017 Aug;24(8):2371–8.

99. Hamada T, Nakai Y, Isayama H, Yasunaga H, Matsui H, Takahara N, et al. Progression-free survival as a surrogate for overall survival in first-line chemotherapy for advanced pancreatic cancer. Eur J Cancer Oxf Engl 1990. 2016 Sep;65:11–20.

100. Colloca G, Venturino A, Guarneri D. Analysis of Response-Related and Time-to-event Endpoints in Randomized Trials of Gemcitabine-Based Treatment Versus Gemcitabine Alone as First-Line Treatment of Patients With Advanced Pancreatic Cancer. Clin Colorectal Cancer. 2016 Sep;15(3):264–76.

101. Dabbous O, Wight C, Crathorne L, Qadan A. A systematic review examining the relationship between progression-free survival and overall survival in adults with untreated metastatic pancreatic cancer. J Clin Oncol Conf 2017 Annu Meet Am Soc Clin Oncol ASCO U S. 2017;35(15 Supplement 1) (no pagination).

102. Wilkerson J, Fojo T. Progression-free survival is simply a measure of a drug’s effect while administered and is not a surrogate for overall survival. Cancer J Sudbury Mass. 2009 Oct;15(5):379–85.

103. Amir E, Seruga B, Kwong R, Tannock IF, Ocaña A. Poor correlation between progression-free and overall survival in modern clinical trials: are composite endpoints the answer? Eur J Cancer Oxf Engl 1990. 2012 Feb;48(3):385–8.

104. Kaufman H, Schwartz L, William W, Sznol M, Del Aguila M, Whittington C, et al. Evaluation of clinical endpoints as surrogates for overall survival in patients treated with immunotherapies. J Clin Oncol Conf 2017 Annu Meet Am Soc Clin Oncol ASCO U S. 2017;35(15 Supplement 1) (no pagination).

105. Tan A, Porcher R, Crequit P, Ravaud P, Dechartres A. Differences in Treatment Effect Size Between Overall Survival and Progression-Free Survival in Immunotherapy Trials: A Meta-Epidemiologic Study of Trials With Results Posted at ClinicalTrials.gov. J Clin Oncol Off J Am Soc Clin Oncol. 2017 May 20;35(15):1686–94.

106. Mushti SL, Mulkey F, Sridhara R. Evaluation of Overall Response Rate and Progression-Free Survival as Potential Surrogate Endpoints for Overall Survival in Immunotherapy Trials. Clin Cancer Res Off J Am Assoc Cancer Res. 2018 May 15;24(10):2268–75.

107. Nie R-C, Chen F-P, Yuan S-Q, Luo Y-S, Chen S, Chen Y-M, et al. Evaluation of objective response, disease control and progression-free survival as surrogate end-points for overall survival in anti-programmed death-1 and anti-programmed death ligand 1 trials. Eur J Cancer Oxf Engl 1990. 2019 Jan;106:1–11.

108. Halabi S, Rini B, Escudier B, Stadler WM, Small EJ. Progression-free survival as a surrogate endpoint of overall survival in patients with metastatic renal cell carcinoma. Cancer. 2014 Jan 1;120(1):52–60.

109. Delea TE, Khuu A, Heng DY, Haas T, Soulières D. Association between treatment effects on disease progression end points and overall survival in clinical studies of patients with metastatic renal cell carcinoma. Br J Cancer. 2012 Sep 25;107(7):1059–68.

110. Johnson KR, Liauw W, Lassere MND. Evaluating surrogacy metrics and investigating approval decisions of progression-free survival (PFS) in metastatic renal cell cancer: a systematic review. Ann Oncol Off J Eur Soc Med Oncol. 2015 Mar;26(3):485–96.

111. Petrelli F, Barni S. Surrogate end points and postprogression survival in renal cell carcinoma: an analysis of first-line trials with targeted therapies. Clin Genitourin Cancer. 2013 Dec;11(4):385–9.

112. Savina M, Litière S, Italiano A, Burzykowski T, Bonnetain F, Gourgou S, et al. Surrogate endpoints in advanced sarcoma trials: a meta-analysis. Oncotarget. 2018 Oct 2;9(77):34617–27.

113. Zer A, Prince RM, Amir E, Abdul Razak A. Evolution of Randomized Trials in Advanced/Metastatic Soft Tissue Sarcoma: End Point Selection, Surrogacy, and Quality of Reporting. J Clin Oncol Off J Am Soc Clin Oncol. 2016 May 1;34(13):1469–75.

114. Flaherty KT, Hennig M, Lee SJ, Ascierto PA, Dummer R, Eggermont AMM, et al. Surrogate endpoints for overall survival in metastatic melanoma: a meta-analysis of randomised controlled trials. Lancet Oncol. 2014 Mar;15(3):297–304.

115. Petrelli F, Coinu A, Cabiddu M, Borgonovo K, Ghilardi M, Lonati V, et al. Early analysis of surrogate endpoints for metastatic melanoma in immune checkpoint inhibitor trials. Medicine (Baltimore). 2016 Jun;95(26):e3997.

116. Sjoquist KM, Lord SJ, Friedlander ML, John Simes R, Marschner IC, Lee CK. Progression-free survival as a surrogate endpoint for overall survival in modern ovarian cancer trials: a meta-analysis. Ther Adv Med Oncol. 2018;10:1758835918788500.

117. Colloca G, Venturino A. Trial-level analysis of progression-free survival and response rate as end points of trials of first-line chemotherapy in advanced ovarian cancer. Med Oncol Northwood Lond Engl. 2017 May;34(5):87.

118. Moriwaki T, Yamamoto Y, Gosho M, Kobayashi M, Sugaya A, Yamada T, et al. Correlations of survival with progression-free survival, response rate, and disease control rate in advanced biliary tract cancer: a meta-analysis of randomised trials of first-line chemotherapy. Br J Cancer. 2016 Apr 12;114(8):881–8.

119. Han K, Ren M, Wick W, Abrey L, Das A, Jin J, et al. Progression-free survival as a surrogate endpoint for overall survival in glioblastoma: a literature-based meta-analysis from 91 trials. Neuro-Oncol. 2014 May;16(5):696–706.

120. Imaoka H, Sasaki M, Takahashi H, Hashimoto Y, Ohno I, Mitsunaga S, et al. Progression-free survival as a surrogate endpoint in advanced neuroendocrine neoplasms. Endocr Relat Cancer. 2017 Sep;24(9):475–83.

121. Liang F, Zhang S, Wang Q, Li W. Evolution of randomized controlled trials and surrogacy of progression-free survival in advanced/metastatic urothelial cancer. Crit Rev Oncol Hematol. 2018 Oct;130:36–43.

122. Rose PG, Tian C, Bookman MA. Assessment of tumor response as a surrogate endpoint of survival in recurrent/platinum-resistant ovarian carcinoma: a Gynecologic Oncology Group study. Gynecol Oncol. 2010 May;117(2):324–9.

123. Abdel-Rahman O. Surrogate end points for overall survival in trials of PD-(L)1 inhibitors for urinary cancers: a systematic review. Immunotherapy. 2018;10(2):139–48.
